# Expression of calbindin-D28k and its regulation by estrogen in the human endometrium during the menstrual cycle

**DOI:** 10.1186/1477-7827-9-28

**Published:** 2011-03-02

**Authors:** Hyun Yang, Tae-Hee Kim, Hae-Hyeog Lee, Kyung-Chul Choi, Yeon-pyo Hong, Peter CK Leung, Eui-Bae Jeung

**Affiliations:** 1 Laboratory of Veterinary Biochemistry and Molecular Biology, College of Veterinary Medicine, Chungbuk National University, Cheongju, Chungbuk 361-763, Republic of Korea; 2 Department of Obstetrics and Gynecology, College of Medicine, Soonchunhyang University, Bucheon 420-767, Republic of Korea; 3 Department of Preventive Medicine, College of Medicine, Chung-Ang University, Seoul 156-756, Republic of Korea; 4 Department of Obstetrics and Gynecology, Faculty of Medicine, University of British Columbia, Vancouver, British Columbia, Canada

## Abstract

Human endometrium resists embryo implantation except during the 'window of receptivity'. A change in endometrial gene expression is required for the development of receptivity. Uterine calbindin-D28k (CaBP-28k) is involved in the regulation of endometrial receptivity by intracellular Ca2+. Currently, this protein is known to be mainly expressed in brain, kidneys, and pancreas, but potential role(s) of CaBP-28k in the human uterus during the menstrual cycle remain to be clarified. Thus, in this study we demonstrated the expression of CaBP-28k in the human endometrium in distinct menstrual phases. During the human menstrual cycle, uterine expression levels of CaBP-28k mRNA and protein increased in the proliferative phase and fluctuated in these tissues, compared with that observed in other phases. We assessed the effects of two sex-steroid hormones, 17beta-estradiol (E2) and progesterone (P4), on the expression of CaBP-28k in Ishikawa cells. A significant increase in the expression of CaBP-28k mRNA was observed at the concentrations of E2 (10(-9 to -7) M). In addition, spatial expression of CaBP-28k protein was detected by immunohistochemistry. CaBP-28k was abundantly localized in the cytoplasm of the luminal and glandular epithelial cells during the proliferative phases (early-, mid-, late-) and early-secretory phase of menstrual cycle. Taken together, these results indicate that CaBP-28k, a uterine calcium binding protein, is abundantly expressed in the human endometrium, suggesting that uterine expression of CaBP-28k may be involved in reproductive function during the human menstrual cycle.

## Background

Intracellular calcium binding proteins (calbindins) are critical for regulating the availability of calcium ions (Ca^2+^) within cells. There are two types of cytosolic calbindins, calbindin-D_9k _(CaBP-9k) and calbindin-D_28k_(CaBP-28k), which are cytosolic proteins differentially regulated by steroid hormones in the uterus [1,2]. In addition to its traditional role in extracellular calcium homeostasis [3], vitamin D influences a broad range of cellular events, ranging from oncogene expression [4] and immunoregulation [5] to cellular differentiation [6,7] and intracellular calcium metabolism [8]. Previous studies have documented that vitamin D deficiency decreases fertility in female rats [9-11], and vitamin D-dependent calcium-binding proteins were discovered in reproductive tissues: CaBP-9k in the uterus of rats [12-14], and the larger CaBP-28k in the uterus of domestic fowl [15,16].

The expression of CaBP-9k and CaBP-28k in reproductive tissues is affected by steroid hormones [12,14,15,17]. For example, the expression of CaBP-9k mRNA in the mouse uterus was significantly increased by treatment with P4 and E2 plus P4, but not by E2 alone [18,19]. Conversely, rat uterine CaBP-D9k mRNA was induced only by E2 [20]. In hens, uterine expression of the larger calbindin-D_28k _increased by the addition of testosterone [15].

Embryo implantation is a complex process involving interactions between the blastocyst and the uterus. A successful implantation requires the development of the blastocyst stage, its escape from the zonapellucida, and the establishment of a receptive uterus [21]. The primate endometrium undergoes certain hormone-dependent changes during a particular time window within the preimplantation phase that prepares it to receive the growing blastocyst [22-24]. A complex interaction between effectors, including steroid hormones, growth factors, and cytokines, regulates development of the "receptivity" state of the uterine epithelium [25-28].

The action of calcium ions in female reproductive organs has also been widely studied for several decades. It has been suggested that calcium ions are involved in uterine smooth muscle contraction and fetal implantation [1,29,30]. Additionally, the balance of calcium ions during uterine contraction and relaxation is extremely important throughout pregnancy and during labor. However, the mechanism of regulation of calcium levels in uterine tissue remains largely unknown. In the present model of calcium flux in uterine tissue, calcium ions flow into the cytoplasm through ion transport proteins, exchangers, or calcium-binding proteins, i.e., CaBP-9k and CaBP-28k. Furthermore, the mechanism and regulation of calcium-related genes in the uterus are not fully characterized, and it is likely that the regulation of calcium ion flux is complex and involves a diverse set of proteins. For instance, implantation in CaBP-9k knockout mice appears to occur normally, while CaBP-9k/CaBP-28k double knockout mice experience failed embryo implantation [28]. These results demonstrate that the expression patterns of CaBP-28k may be involved in reproductive function and fetal implantation during the menstrual cycle in female mice.

In our previous studies, the expression of uterine CaBP-28k mRNA and protein in mice during the estrus cycle is regulated by sex-steroid hormones [18,31]. However, the role of CaBP-28k in the human uterus has yet to be fully characterized. It is likely that CaBP-28k is functionally important in female reproductive organs. We examined the effect of steroid hormones E2 and P4 on the expression of CaBP-28k in the human endometrial tissues during the different stages of the menstrual cycle and in Ishikawa endometrial cancer cells. In addition, we also examined the intracellular localization of uterine CaBP-28k in human female endometrial tissue.

## Methods

### Materials

17β-Estradiol (E2), progesterone (P4), and mifepristone (RU486) were obtained from Sigma-Aldrich Corporation (St. Louis, MO). ICI 182 780 was purchased from TOCRIS (Avonmouth, UK).

### Endometrial tissue

Human endometrial tissues were collected by curettage from women (the ages of 28-45 years) undergoing hysteroscopy for investigation of tubal p"36atency or tubal ligation. Endometrial tissues were classified according to the most recent menstrual period, and histology was performed according to the criteria of Noyes et al. [32]. Approval was given by the Human Ethics Committee at SCH Medical Center (Bucheon, Korea), and signed consent was obtained in every case. Human uterine finally classified samples (total *n *= 42) were divided into seven groups (n = 6 in each period): menstrual, proliferative (early, mid, late), and secretory phase (early, mid, late). Endometrial tissues were transported to the laboratory in buffered neutral formalin (10%) on ice. Tissues were then quickly frozen in liquid nitrogen and stored at -70°C until further use.

### Cell culture and treatment

Ishikawa cells were obtained from Sigma-Aldrich (St. Louis, MO). The cells were grown as monolayer cultures in Dulbecco's Modified Eagle Medium (DMEM; Gibco BRL, Grand Island, NY), supplemented with 10% fetal bovine serum (FBS; Gibco BRL), 100 IU/mL penicillin, and 100 μg/mL streptomycin (Gibco BRL) at 37°C in a humidified atmosphere of 95% O_2 _and 5% CO_2_. To challenge Ishikawa cells to E2 and P4, the cells were plated and grown to 70-80% confluence in six-well plastic tissue culture dishes (NUNC™, Roskilde, Denmark). In order to ensure depletion of steroid hormones and growth factors in the cells, the growth medium was replaced with starvation media containing phenol red-free DMEM with 5% dextran-coated charcoal-stripped FBS, 100 IU/mL penicillin, and 100 μg/mL streptomycin, as described previously [33]. The Ishikawa cells were maintained on starvation media for 3 days before exposure to three concentrations of E2 (10^-9 ^M, 10^-8 ^M, and 10^-7 ^M) and P4 (10^-7 ^M, 10^-6 ^M, and 10^-5 ^M). To further investigate whether ER or PR is involved in the expression of CaBP-28k protein, the cells were pretreated with ICI 182 780 (10^-6 ^M) or mifepristone (10^-6 ^M) for 60 min prior to the treatment with E2 (10^-8 ^M) or P4 (10^-6 ^M). Each chemical was dissolved in DMSO and added to the starvation media with the final DMSO concentration being 0.1%. DMSO alone was used as a negative control. Ishikawa cells were harvested 48 h after treatment with E2 or P4 to measure mRNA levels, and whole cells were harvested for mRNA and Western blot analyses. All experiments were performed in triplicate (group n = 3).

### Total RNA extraction and quantitative real-time PCR

Endometrial tissues were transported, rapidly excised, and washed in cold, sterile NaCl (0.9%). Total RNA was prepared with Trizol reagent (Invitrogen, Carlsbad, CA), and the concentration of RNA was determined by absorbance at 260 nm. Total RNA (1 μg) was reverse transcribed into first-strand cDNAs using Moloney murine leukemia virus (MMLV) reverse-transcriptase (Invitrogen, Carlsbad, CA) and random primers (9-mers; Takara Bio Inc., Otsu, Shiga, Japan). Two microliters of cDNA template was added to 10 μL of 2× SYBR Premix Ex Taq (TaKaRa Bio) and 10 pmol of each specific primer. The reactions were carried out for 40 cycles according to the following parameters: denaturation at 95°C for 30 s, annealing at 58°C for 30 s, and extension at 72°C for 45 s. The oligonucleotide primers for CaBP-28k were 5'- AGT GGT TAC CTG GAA GGA AAG G -3' (sense) and 5'- AGC AGG AAA TTC TCT TCT GTG G -3' (antisense). The primers for GAPDH were 5'- GGT GTG AAC CAT GAG AAG TAT GAC -3' (sense) and 5'- AGT AGA GGC AGG GAT GAT GTT CT -3' (antisense). Fluorescence intensity was measured at the end of the extension phase of each cycle. The threshold value for the fluorescence intensity of all samples was set manually. The reaction cycle at which PCR products exceeded this fluorescence intensity threshold was identified as the threshold cycle [34] in the exponential phase of the PCR amplification. The expression of calbindin-D_28k _was quantified against that of GAPDH. Relative quantification was based on the comparison of CT [cycle threshold] at a constant fluorescent intensity. The amount of transcript is inversely related to the magnitude of observed CT, and for every two-fold dilution in the transcript, CT is expected to increase by one. Relative expression was calculated using the equation *R *= 2^- [ΔCT sample - ΔCT control] ^[26]. To determine a normalized arbitrary value for each gene, every obtained value was normalized to that of GAPDH.

### Reverse transcription polymerase chain reaction (RT-PCR)

Total RNA was extracted and cDNA synthesized as described in the preceding paragraph. RT-PCR was performed as described by [35]. Denaturation was performed at 95°C for 30 s, annealing at 58°C for 30 s (for CaBP-28k) or at 55°C for 30 s (for GAPDH), and extension at 72°C for 30 s. Cycling kinetics were performed using 20, 25, and 30 cycles, to ensure linearity of PCR product detection, and the final PCR condition was 30 cycles for CaBP-28k or 25 cycles for GAPDH. Eight microliters of PCR products was loaded on 2% agarose gel and stained with ethidium bromide following electrophoresis. The intensity of the PCR bands was determined directly by scanning the agarose gel and was analyzed using the molecular analysis program version 4.5.1 (Gel Doc 1000, Bio-Rad, Hercules, CA).

### Western blot analysis

Endometrial tissues were transported, rapidly excised, and washed in cold sterile 0.9% NaCl solution. Protein was extracted with Pro-prep (iNtRON Bio Inc, Sungnam, Kyungki-Do, Korea) according to the manufacturer's instructions. The cells were harvested, washed two times with ice-cold PBS, and then resuspended in 20 mM Tris-HCl buffer (pH 6.4) containing protease inhibitors (0.1 mM phenylmethylsulfonyl fluoride, 5 μg/mL pepstatin A, and 1 μg/mL chymostatin). Whole cell lysate was prepared using 20 strokes of a Dounce homogenizer, followed by centrifugation at 13,000*g *for 20 min at 4°C. Proteins (70 μg per lane) were separated on a 10% SDS-polyacrylamide gel electrophoresis (PAGE) gel and transferred to a polyvinylidene fluoride transfer membrane (Perkin Elmer Co., Wellesley, MA) in a TransBlot Cell (TE-22, Hoefer Inc, San Francisco, CA) according to the manufacturer's protocol. The resulting blot was blocked in TBS-T (Tris-Buffered Saline Tween-20) containing 5% skim milk for 60 min and then incubated with primary antibody: CaBP-28k (goat-polyclonal, 1:500, Santa Cruz, CA, USA) or GAPDH (mouse-monoclonal, 1:2000, Assay Design Inc, Ann Abor, MI). After being washed in buffer, the membranes were incubated with the appropriate horseradish peroxidase-conjugated secondary antibodies (anti-goat, 1:2000 or anti-mouse, 1:5000, Santa Cruz, CA) for 1 h at room temperature (RT). After being washed, the blots were developed by incubation in ECL chemiluminescence reagent (Santa Cruz, CA) and subsequently exposed to Biomax™ Light film (Kodak) for 1-5 min. Signal specificity was confirmed by blotting in the absence of primary antibody, and bands were normalized to GAPDH-immunoreactive bands visualized in the same membrane after stripping. Density measurements for each band were performed with NIH ImageJ software. Background samples from an area near each lane were subtracted from each band to obtain mean band density.

### Immunohistochemical staining

Localization of CaBP-28k protein was examined by immunohistochemistry. Endometrial tissues were embedded in paraffin, before sections (5 μm) were deparaffinized in xylene and hydrated in descending grades of ethanol. Endogenous peroxidase activity was blocked with 3% hydrogen peroxide in TBS-T for 30 min. Nonspecific reactions were blocked by incubating the sections in 10% normal goat serum for 2 h at RT. The sections were subsequently incubated at RT for 4 h with a polyclonal goat antibody directed against CaBP-28k (diluted 1:300; Santa Cruz, CA, USA) dissolved in 10% normal goat serum. After being washed with TBS-T, the sections were incubated with a biotinylated secondary antibody (goat IgG, Vector Laboratories, Burlingame, CA) for 30 min at 37°C and then incubated with ABC-Elite for 30 min at 37°C. Diaminobenzidine (DAB; Sigma) was used as a chromogen, and the sections were counterstained with hematoxylin, followed by mounting in Canada balsam.

### Data analysis

Data were presented as the means ± SEM and analyzed by one-way analysis of variance (ANOVA), followed by Tukey's multiple comparison test. Statistical analysis was performed using Prism Graph Pad (v4.0; GraphPad Software Inc., San Diego, CA, USA). *P *< 0.05 was considered to be statistically significant.

## Results

### Pattern of endometrial calbindin-D_28k _expression

**Figure 1:**
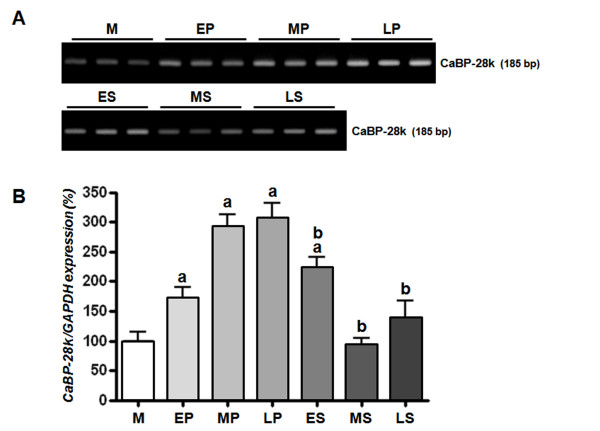
**Pattern of endometrial CaBP-28k mRNA expression**. Endometrial tissues were divided into 7 groups (M: menstrual phase, EP: early-proliferative phase, MP: mid-proliferative phase, LP: late-proliferative phase, ES: early-secretory phase, MS: mid-secretory phase, LS: late-secretory phase) based on the endometrial tissues classified according to the last menstrual period and histology according to the criteria of Noyes *et al *[32]. Uterine calbindin-D_28k _mRNA expression during the menstrual cycle was examined by (**A**: RT-PCR; **B**: real-time PCR). A significant increase in calbindin-D_28k _expression level was observed during menstrual cycle *(^a^P < 0.05 vs. M, ^b^P < 0.05 vs. MP or LP)*.

**Figure 2:**
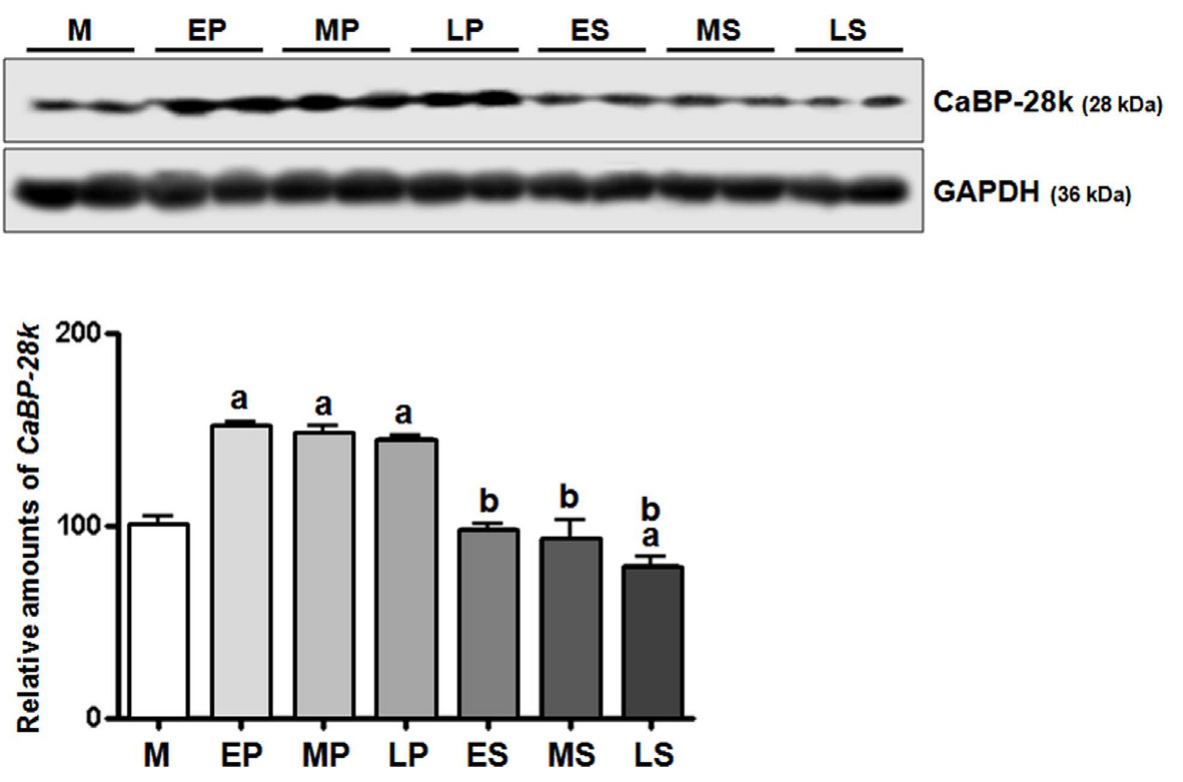
**Pattern of endometrial CaBP-28k protein expression**. Endometrial tissues were divided into 7 groups (M: menstrual phase, EP: early-proliferative phase, MP: mid-proliferative phase, LP: late-proliferative phase, ES: early-secretory phase, MS: mid-secretory phase, LS: late-secretory phase) based on the endometrial tissues classified according to the last menstrual period and histology according to the criteria of Noyes *et al *[32]. Uterine CaBP-28k protein expression during the menstrual cycle was examined by WESTERN BLOT. A significant increase in calbindin-D_28k _expression level was observed during menstrual cycle *(^a^P < 0.05 vs. M, ^b^P < 0.05 vs. MP or LP)*.

To investigate the expression of endometrial CaBP-28k during the menstrual cycle, we divided the endometrial tissues into seven groups [menstrual phase, proliferative phase (early, mid, late), and secretory phase (early, mid, late) according to the most recent menstrual period, and histology according to the criteria of Noyes et al. [32]. In normalization to GAPDH, CaBP-28k mRNA levels in the proliferative phase (early, mid, late) and secretory phase (early) were induced up to 1.5-, 2.5-, 2.5-, and 2.0-fold higher than in the menstrual phase, respectively (Figure 1). This induced expression of CaBP-28k was significantly decreased in secretory phases (early, mid, and late) compared to that in proliferative phases (mid and late phases). In parallel with mRNA levels, the protein levels of CaBP-28k were higher in the proliferative phase (early-, mid-, late-) than in the other phases (Figure 2). This induced protein level of CaBP-9k was also reduced in secretory phases compared to that of proliferative phases as shown in Figure 2.

### Effect of sex-steroid hormone E2 and P4 on regulation of calbindin-D_28k _expression in Ishikawa cells

**Figure 3:**
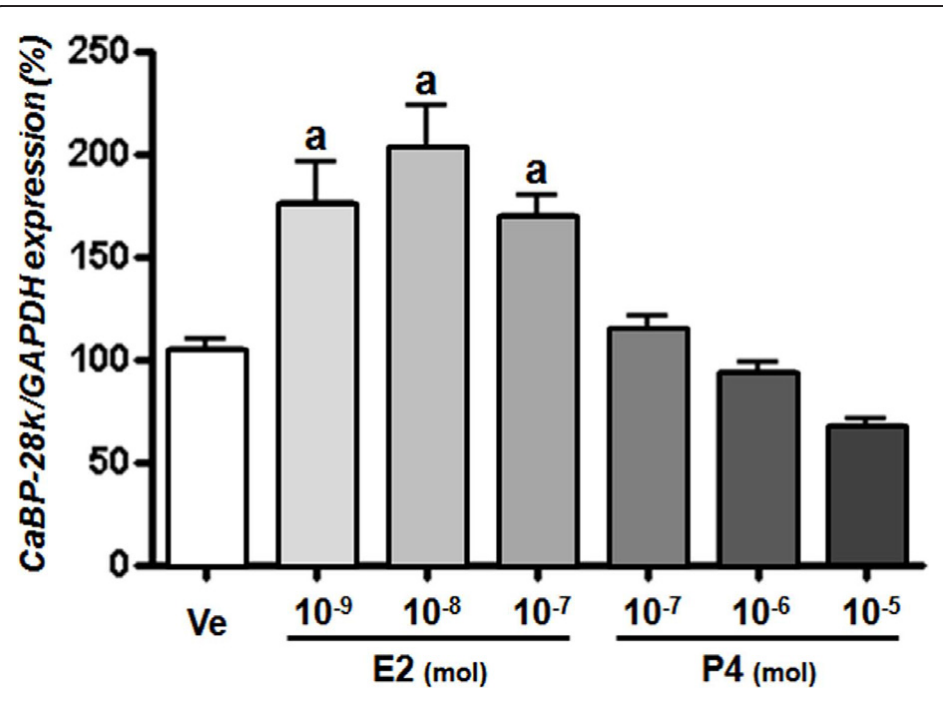
**Effect of sex-steroid hormone E2 and P4 on regulation of CaBP-28k mRNA expression**. The cells were treated with only DMSO (Ve, negative control), E2 (10^-9 to -7 ^mol), P4(10^-7 to -5 ^mol) for 2 days. Graphs represent the analysis of real-time PCR data. *^a^P < 0.05 *indicate a significant difference in CaBP-28k expression level compared to a negative control.

**Figure 4:**
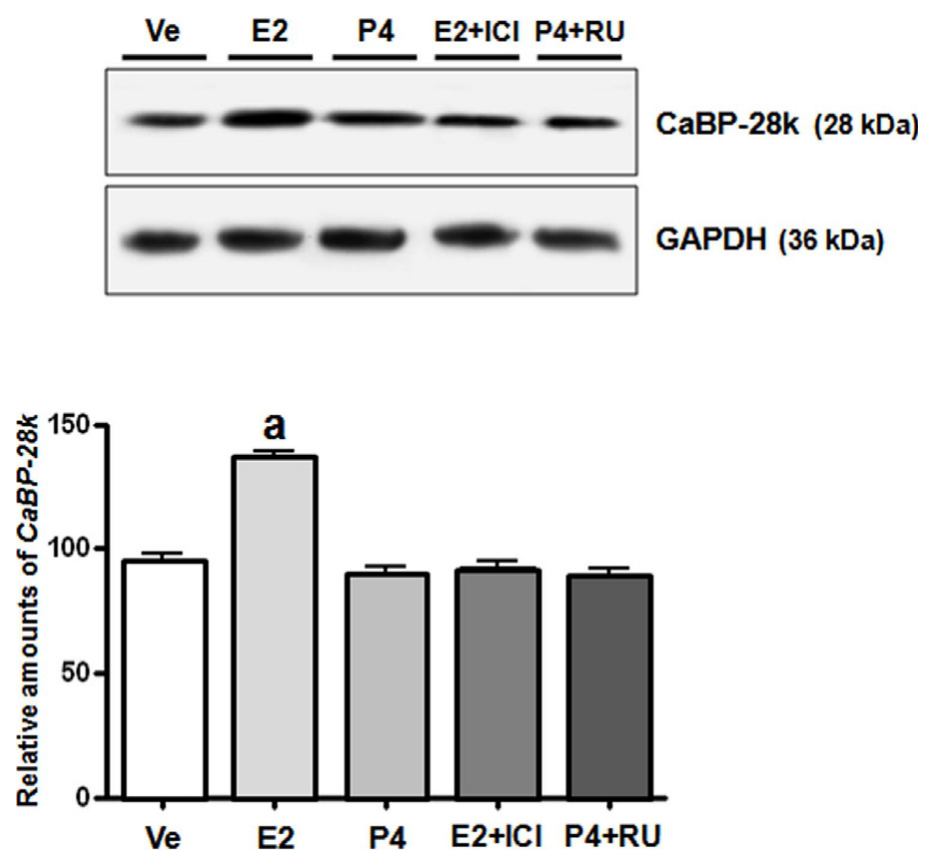
**Effect of sex-steroid hormones and ER and PR antagonists on regulation of CaBP-28k protein expression**. The cells were treated with only DMSO (Ve, negative control), E2 (10^-8^M), P4 (10^-6^M) and/or ICI (ICI 182,780: 10^-6^M), RU (RU486: 10^-6^M) for 2 days and harvested 24 h following the final injection. Graphs represent the analysis of Western blot data. *^a^P < 0.05 *indicate a significant difference in CaBP-28k expression level compared to a negative control.

We assessed the effects of sex-steroid hormones E2 and P4 on the regulation of CaBP-28k in Ishikawa cells. A panel of chemicals comprising E2 and P4 were applied to Ishikawa cells at increasing concentrations (E2: 10^-9 ^M to 10^-7 ^M, and P4: 10^-7 ^M to 10^-5^M). As shown in Figure 3, a significant increase in the expression of CaBP-28k mRNA was observed in the cells treated with E2 (10^-9 ^M to 10^-7^M). Compared to vehicle (negative) controls, CaBP-28k mRNA levels were increased up to 2.0- and 2.5-fold, respectively (Figure 3). Also, we elucidated the involvement of the estrogen receptor (ER) in the E2-induced CaBP-28k increase in Ishikawa cells by treating the cells with an ER antagonist or PR antagonist (ICI and mifepristone, respectively) for 60 min prior to the treatment with E2 (10^-8 ^M) or P4 (10^-6 ^M). Pretreatment with an ER antagonist completely reversed the E2-induced increase in CaBP-28k expression (Figure 4), suggesting that ERs are involved in the E2-mediated regulation of CaBP-28k expression in Ishikawa cells. Furthermore, after pretreatment with a PR antagonist, the P4-induced CaBP-28k expression was not changed, indicating that PRs are not involved in the expression of CaBP-28k protein in Ishikawa cells.

### Localization of calbindin-D_28k _expression in human endometrial tissue

**Figure 5:**
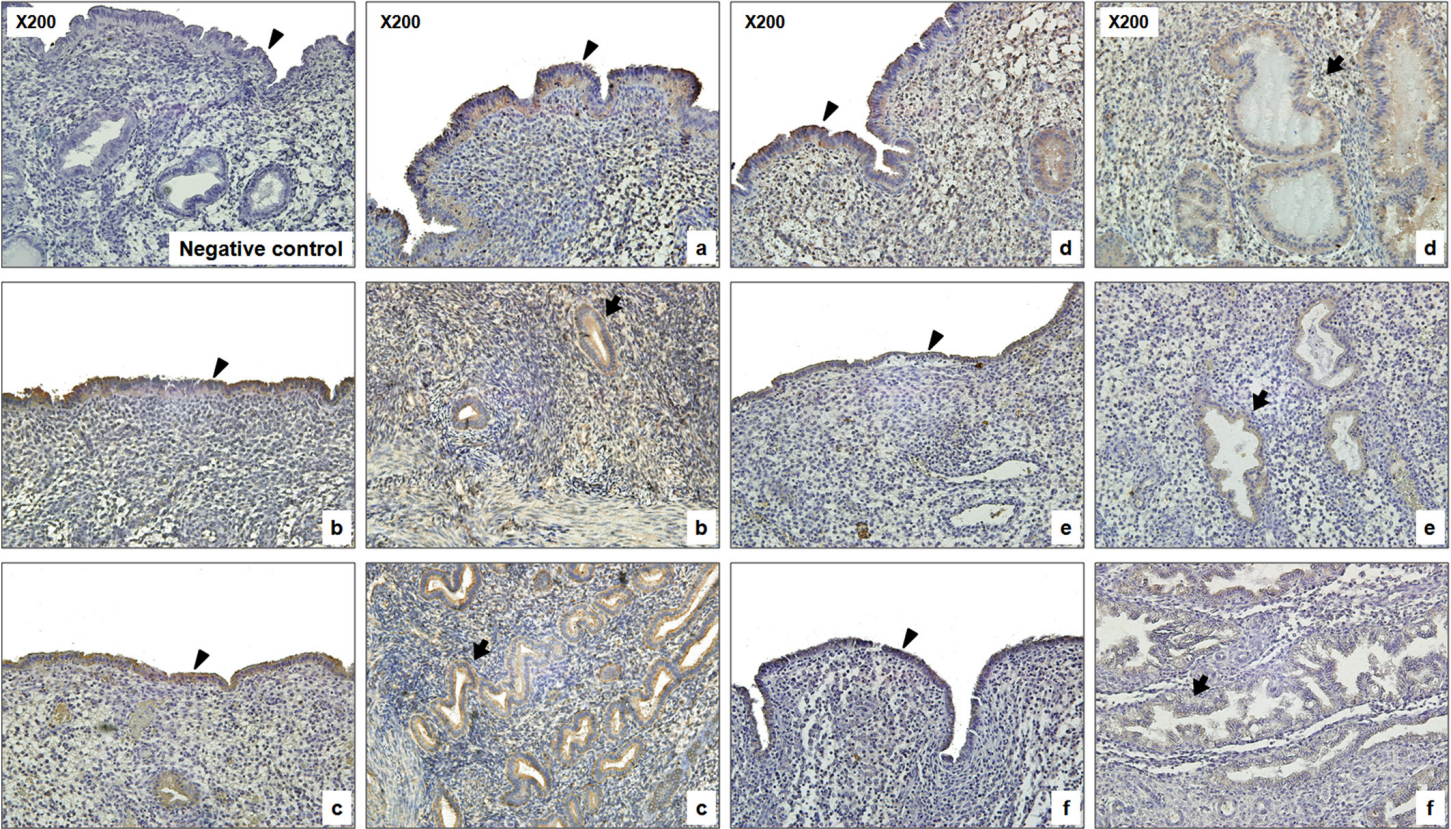
**Localization of CaBP-28k protein in the human endometrium**. Immunohistochemistry was performed to detect CaBP-28k protein in different phases in the uterine sections during the menstrual cycle (a, early-proliferative phase; b, mid-proliferative phase; c, late-proliferative phase; d, early-secretory phase; e, mid-secretory phase; f, late-secretory phase) as described in Materials and Methods. Arrowheads **→ **endometrial epithelial cells, arrows **→ **endometrial glandular cells. Arrows and arrowheads indicate CaBP-28k-positive regions.

To assess the spatial pattern of expression of CaBP-28k in the endometrial tissue, we analyzed tissue sections at different stages of the menstrual phases (early-, mid-, late-proliferative phase or secretory phase) by immunohistochemistry using an anti-CaBP-28k antibody. Endometrial CaBP-28k was highly expressed in cytoplasm of endometrial epithelial cells in early-, mid-, late-proliferative phase compared to secretory phases. Additionally, in the early-secretory phase, CaBP-28k was weakly expressed in the endometrial epithelial cells as compared to the glandular epithelial cells (Figure 5).

## Discussion

We have previously determined that calcium-related proteins are regulated by sex-steroid hormones in the uterus of rodents [26,31,36,37]. It is well known that the status of the uterine cavity is important for successful implantation and is influenced by the secretory activity of the glandular epithelium [15,38,39]. In this study, CaBP-28k mRNA and protein were expressed in human endometrial tissues. The levels of calcium-related proteins fluctuated in E2-predominant stages [20,26,37]. CaBP-28k mRNA and protein were expressed at these stages [proliferative (early, mid, late) and secretory (only early) phase], followed by a decline in the secretory phase (mid-, late-) of the human endometrium, as demonstrated in this study. This result is in agreement with the pattern of CaBP-28k expression in the uterus of both mice and humans [28]. Therefore, these results indicate that this parallel pattern of CaBP-28k expression may be also involved in fetal implantation in humans.

Moreover, the level of CaBP-28k mRNA was increased by E2 (E_2_, 10^-9 ^M to 10^-7 ^M) in human endometrial cancer cells (Ishikawa cells) as shown in this study. Sex-steroid hormones can induce changes in the structure and function of the uterus and regulate menstrual cycle progression. To examine the effect of these hormones on uterine CaBP-28k expression, we treated Ishikawa cells with E2 (10^-8 ^M) or P4 (10^-6 ^M). Treatment with E2 resulted in increased uterine expression of CaBP-28k mRNA and protein, while P4 did not alter CaBP-28k expression. The expression of several calcium-related proteins is altered in an E2-dependent [31,37] or P4-dependent [26,36] manner in the estrus cycle. It is particularly interesting that calcium-related proteins are expressed during the estrus cycle, due to the wide variation in regulation of calcium-related protein expression in the uterus across species. Calcium-related proteins, which are crucial for early implantation, are also expressed in the endometrial layer [27,28,39]. However, it is still unclear why the uterine luminal epithelium increases Ca^2+ ^uptake during implantation. In what aspect would this uptake facilitate blastocyst attachment at implantation? It is known that, for proper blastocyst implantation in human and mice, there must be interaction between adhesion-competent trophoblast cells and endometrial extracellular matrix components. The accumulation of integrin receptors for fibronectin on the blastocyst surface enables it to bind to and penetrate the extracellular matrix (ECM) [40], but only when the blastocyst has reached an adhesion-competent state. Recently, it has been shown that the development of an adhesion-competent embryo is facilitated by heparin-binding epidermal growth factor (EGF)-like growth factor (HB-EGF) and that this process is dependent on calcium influx from extracellular sources [41]. The uterine epithelium is a barrier to interstitial blastocyst implantation, and it is also an important participant in the maternal-embryonic dialogue. During the periimplantation period, uterine epithelial cells produce HB-EGF [42] and the uterine glands secrete calcitonin [43], two paracrine or juxtacrine agents that are each capable of accelerating blastocyst differentiation. It is intriguing that the biological activity of both factors is dependent on Ca^2+ ^signaling [44], which appears to regulate preimplantation embryogenesis beginning as early as the maturing oocyte [45,46]. These data suggest that a possible source of this external calcium needed for HB-EGF-mediated differentiation of the blastocyst to an adhesion-competent state is from stores in the luminal epithelium of the endometrium.

As previously shown, the initial up-regulation of CaBP-28k increases the storage capacity for Ca^2+ ^in luminal epithelial cells, and the subsequent specific down-regulation leads to an increase in free Ca^2+ ^concentration [28]. In mammalian enterocytes, free Ca^2+ ^ions are bound to cytosolic CaBP-9k and transferred across the cells by facilitated diffusion [47]. This transport of Ca^2+ ^by CaBP-9k helps to maintain homeostasis by keeping intracellular Ca^2+ ^ion concentrations below 10^-7 ^M, preventing premature cell death by apoptosis. Thus, it can be speculated that the role of CaBP-28k protein in the uterine luminal epithelium is also to enhance Ca^2+ ^uptake by increasing the cell-buffering capacity and stimulating the calcium entry mechanism in these cells. This release may trigger apoptosis in these specific epithelial cells because high concentrations of free Ca^2+ ^are reported to cause apoptosis in many different cell types [48] and CaBP-28k is able to inhibit apoptosis in osteoblastic cells [49]. This apoptosis could, in turn, destabilize the epithelial barrier at the implantation site and facilitate trophoblast invasion and implantation.

An immunohistochemistry study revealed that the CaBP-28k protein was highly expressed in the cytoplasm of endometrial epithelial cells in the early-, mid-, late-proliferative compared with other phases, however, it was weakly expressed in endometrial epithelial cells as compared to that in glandular epithelial cells in the early-secretory phase. In the previous study, it was particularly interesting to observe that the regions where the uterine epithelium surrounded the implanting embryo were negative for CaBP-9k mRNA, whereas uniform expression was detected in the entire luminal epithelium in the regions where no embryo was attached [19]. Although CaBP-9k expression is not detected in human endometrium, either of CaBP-9k or CaBP-28k expression appears to be sufficient to facilitate implantation in mice [27]. We did not examine expression pattern(s) of CaBP-28k in the human uterus during pregnancy, partly because it is difficult to collect uterine samples from pregnant subjects due to ethical issues. Although we established that CaBP-28k expression occurred only during the menstrual cycle, we hypothesized that uterine CaBP-28k may be effectively mediated through maternal-fetal dialogue despite species differences between human and rodents. Thus, we assumed that CaBP-28k expression may be involved in endometrial receptivity during the menstrual cycle in humans.

## Competing interests

The authors declare that they have no competing interests.

## Authors' contributions

HY and TK carried out the overall experiments including molecular experiments. TK, HL and YH provided human endometrial tissues and planed clinical experiments. KC participated in the real-time PCR and immunohistochemical analysis. PL participated in the design of the study, performed the statistical analysis and helped to finalize the manuscript. EJ* designed and coordinated the overall study as a corresponding author and helped to draft the manuscript. All authors read and approved the final manuscript.
